# Progress in the clinical application of allogeneic platelet-rich plasma for diabetic foot treatment

**DOI:** 10.3389/fendo.2025.1694691

**Published:** 2025-10-24

**Authors:** Huijuan Qin, Hui Yang, Jing Wang, Dong Jiang, Yinchen Chen, Wei Wang, Aiping Wang

**Affiliations:** Department of Endocrinology, Nanjing Junxie Hospital, Nanjing, China

**Keywords:** allogeneic platelet-rich plasma, autologous platelet-rich plasma, diabetic foot, chronic wound, diabetes mellitus

## Abstract

Due to peripheral neuropathy and varying degrees of vascular disease, patients with diabetes often suffer from foot ulcers that are challenging to heal and may even progress to gangrene. In recent years, autologous platelet rich plasma (au-PRP) gel has been widely used in the treatment of chronic ulcers, including diabetes foot. However, its limitations have become increasingly evident, such as difficulties in collecting sufficient blood from patients, deficiencies in platelet count and/or function among individuals with diabetes, and high costs associated with individually required equipment. Allogeneic platelet rich plasma (al-PRP) offers similar anti-inflammatory, anti-infective, and growth promoting effects while addressing these drawbacks. Notably, al-PRP can be mass-produced into ready-to-use products, simplifying clinical applications and significantly reducing costs. This article conducted a comprehensive analysis of the application mechanism, necessity, effectiveness and safety, preparation and application methods of al-PRP in the treatment of diabetic foot ulcers, it showed that al-PRP has a promising application prospect in the treatment of diabetic foot.

## Introduction

Chronic wounds, including diabetic foot ulcers, have emerged as a global health concern due to the aging population and the rising prevalence of obesity, diabetes, and cardiovascular diseases (1).The complexity inherent in managing chronic wounds has made promoting soft tissue repair and wound healing a prominent research focus. According to the latest edition of the International Diabetes Federation (IDF), approximately 537 million adults worldwide are currently living with diabetes mellitus, a number projected to rise to 783 million by 2045 (2). Diabetic foot and lower limb complications represent a serious chronic health issue, affecting approximately 18.6 million people worldwide annually. Diabetic foot ulcers (DFUs) and amputations significantly diminish patients’ quality of life and can even result in premature death (3). Comprehensive pathway management of DFUs remains the best practice (4). It includes debridement, decompression, infection control, and optimization of metabolism and blood supply, and involves treatments such as negative pressure wound therapy, advanced dressings, vascular reconstruction, and flap transplantation. However, it has significant issues of cost and accessibility barriers, failing to meet the requirements of low cost and high feasibility.

In 1954, Kingsley first described platelet-rich plasma (PRP) as a blood product with a significantly elevated platelet count. From the 1960s to the 1970s, the improvement of density gradient centrifugation played a crucial role in blood component separation, advancing the development of PRP. In 1987, PRP was first applied in clinical treatment in cardiac surgery and demonstrated favorable efficacy; thereafter, it was gradually adopted in various medical fields such as oral and maxillofacial surgery, and the management of musculoskeletal conditions related to sports injuries. Platelet-rich plasma, a therapeutic technology in regenerative medicine, has been applied in clinical practice. Autologous platelet-rich plasma (au-PRP), derived from a patient’s own whole blood, is a plasma concentrate enriched with platelets, fibrin, bioactive proteins and varying concentrations of leukocytes obtained through centrifugation (5),au-PRP promotes tissue repair by enhancing microcirculation reconstruction, inhibiting local inflammatory responses and reducing apoptosis. However, its application is often limited among patients with multiple comorbidities, such as thrombocytopenia or severe infections, which can compromise the activity and effectiveness of au-PRP. The advent of allogeneic platelet rich plasma (al-PRP) addresses these challenges.

Derived from the platelet concentrate of healthy donor blood, al-PRP releases a significant amount of growth factors and various bioactive substances to support tissue repair and regeneration. In 2007, European researchers reported the first case of using allogeneic platelet-rich plasma for treating delayed fracture union, and this study was published in the journal Injury. Since then, research on the application of allogeneic PRP in fields such as refractory wounds and delayed fracture union has gradually increased. The advantages include ease of preparation, cost-effectiveness, and highly efficacy. This article provides a comprehensive review of the research progress on al-PRP in the treatment of diabetic foot ulcers.

## Mechanism of PRP in the treatment of DFUs

### Anti-inflammatory and anti-infective effects of PRP

Upon activation, platelets in PRP release a spectrum of mediators that promote the recruitment and clustering of macrophages and neutrophils. These effector cells exert direct antimicrobial actions and concurrently shape the local inflammatory milieu (6). They also recognize and remove senescent or injured cells, thereby clearing focal necrotic debris and supporting tissue repair. Macrophage-derived TGF-β drives fibroblast proliferation and their recruitment into the wound bed. In parallel, macrophages and neutrophils provide broad-spectrum clearance of exogenous pathogens in infected wounds through non-specific phagocytosis. Simultaneously, they initiate a cascade of inflammatory responses, including cytokine and chemokine release, upregulation of adhesion molecules, complement activation, and recruitment of additional immune cells to the wound site. Concurrently, macrophages polarize toward the pro-inflammatory M1 phenotype, releasing IL-6 to amplify inflammatory signaling and promote the clearance of necrotic tissue and cellular debris, thereby supporting early wound debridement and defense. Furthermore, macrophages directly influence fibroblasts by secreting growth factors and cytokines (e.g., TGF-β, PDGF, IL-1β) that enhance fibroblast proliferation, migration, and myofibroblast differentiation. These signals increase extracellular matrix synthesis (collagen, fibronectin) and promote wound contraction, thereby accelerating tissue repair and regeneration while laying the groundwork for subsequent remodeling (7). Neutrophils present in al-PRP can release bacteriostatic and antimicrobial factors (e.g., ɑ-defensins, cathelicidins, lactoferrin) and mobilize chemokines such as stromal cell-derived factor-1ɑ (SDF-1ɑ). Alongside other blood cell components (leukocytes and monocytes), these mediators help curtail microbial growth, modulate immune cell trafficking, and temper excessive inflammation. As a result, al-PRP can reduce the expression of pro-inflammatory cytokines (such as IL-1β, IL-6, and TNF-ɑ), supporting a more balanced inflammatory milieu conducive to healing (8).

**Figure 1:**
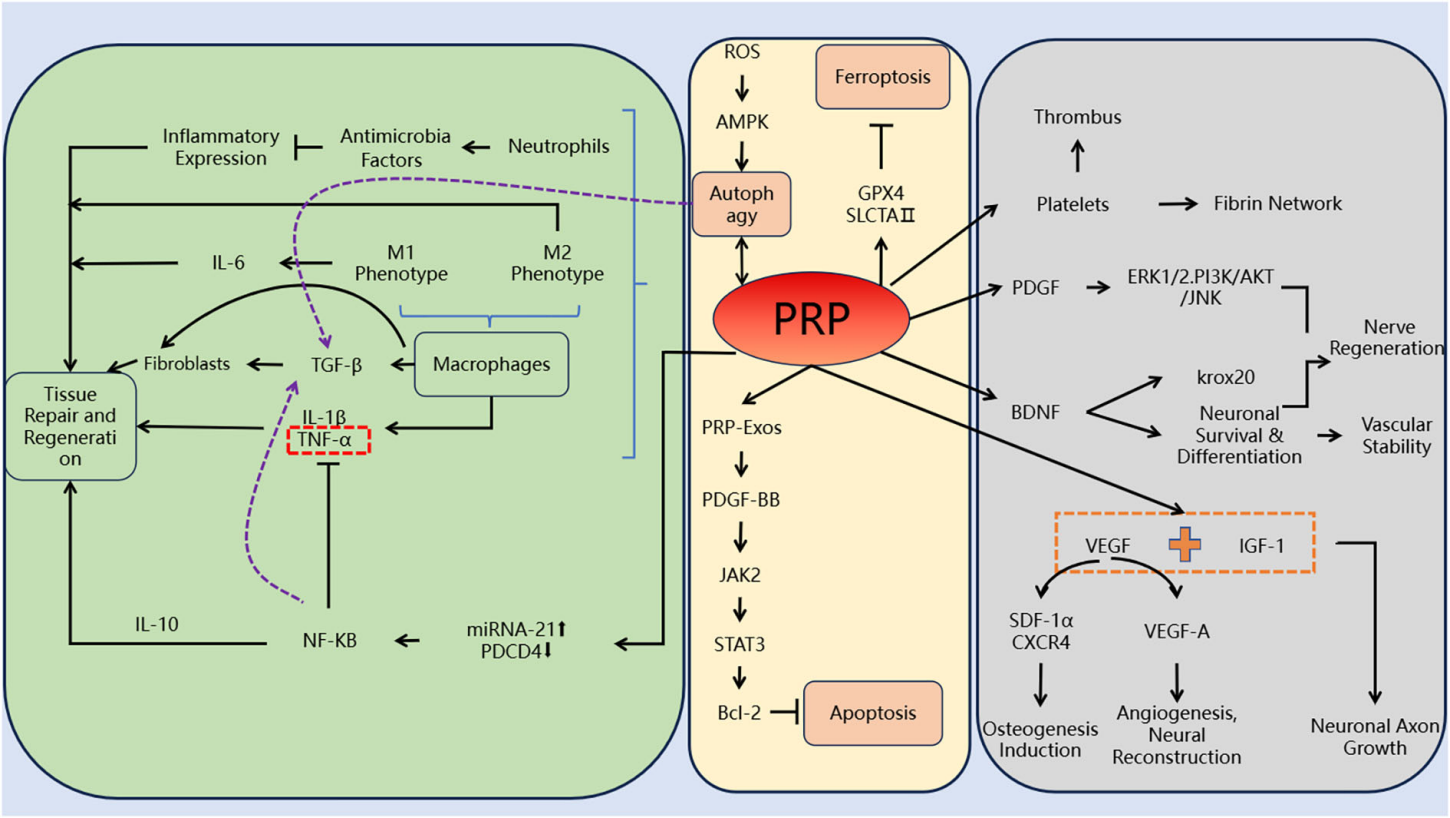
Mechanism of PRP.

The fibronectin-rich fibrillar mesh in PRP sequesters and concentrates platelets and leukocytes, creating a provisional matrix that prolongs and localizes the release of growth factors (e.g., PDGF, TGF-β, VEGF) and cytokines. This scaffold not only enhances cell adhesion and migration but also modulates immune responses by regulating leukocyte activation, macrophage polarization, and complement interactions, thereby fine-tuning inflammation and supporting organized tissue repair (9). Comparative studies indicate that rabbit platelet microbicidal proteins (PMP-1 and thrombin-induced tPMP-1) share high sequence homology with human platelet factor 4 (PF4/CXCL4) and adopt closely similar three-dimensional chemokine-like folds. This structural conservation helps explain their overlapping cationic, heparin-binding, and antimicrobial properties, including membrane-targeting activity against bacteria. Platelets can be activated either by vascular injury signals (thrombin and ADP) or directly by recognition of microorganisms. This activation triggers the release of kinases that chemotactically recruit and activate immune cells, thereby generating inflammatory responses (10). Huang et al. reported that in diabetic ulcer repair, persistent macrophage activation drives elevated proinflammatory cytokines, including TNF-ɑ. Topical platelet-rich plasma (PRP) markedly lowered TNF-ɑ and IL-1β levels in diabetic rat wounds, indicating dampened inflammation and corresponding acceleration of wound closure (11). Moreover, immunomodulatory cues—including IL-4, IL-13, IL-10, glucocorticoids, and immune complexes—can skew macrophages toward an M2 phenotype. M2-polarized macrophages exert anti-inflammatory, regulatory, and tissue-remodeling actions, collectively enhancing the pace of wound repair. Using an *in vitro* diabetic foot model, Li et al. showed that platelet-rich plasma (PRP) attenuates inflammatory signaling while promoting proliferative activity in infected diabetic foot ulcers (12). The anti-infective mechanism has been illustrated within the green box of **Figure 1**.

### Anti-bacterial effects of PRP

Prior work has shown that thrombin-induced platelet microbicidal proteins (tPMPs) secreted by rabbit platelets enhance the bactericidal efficacy of oxacillin and vancomycin against Staphylococcus aureus by lowering the antibiotics’ minimum inhibitory concentrations and delaying microbial growth. Additionally, tPMPs interact with cell surface structures of Candida albicans, disrupting membrane integrity and impairing fungal viability, thereby contributing to antifungal activity (13). A randomized rat study found that PRP treatment significantly increased macrophage colony-stimulating factor expression, CD31, type I collagen, keratinocyte proliferation, and re-epithelialization compared with controls. Moreover, the topical application of PRP effectively reduced inflammation, thereby accelerating wound healing in wounds infected with methicillin-resistant Staphylococcus aureus (MRSA) (14). PRP has shown antimicrobial activity against multidrug-resistant pathogens in diabetic foot ulcers, including MRSA, ESBL-producing Klebsiella pneumoniae, and carbapenem-resistant Pseudomonas aeruginosa. The primary mechanism appears to involve modulation of inflammation via the miRNA-21/PDCD4/NF-κB pathway, leading to reduced IL-6 and TNF-ɑ and increased IL-10 and TGF-β, which collectively promotes an anti-inflammatory, pro-resolving environment conducive to infection control and tissue repair (15).

In a previous animal study, Italian researchers found that an allogeneic platelet-lysate-derived platelet-associated blood derivative could effectively treat cows with acute and chronic clinical mastitis caused by both Gram-positive and Gram-negative bacteria (16). Clinical studies have revealed that in an *in vivo* MRSA-infected skin wound model, al-PRP can shorten the inflammatory cascade response, thereby accelerating the transition to the proliferative phase of wound healing. Combining al-PRP with β-lactam antibiotics significantly reduced MRSA burden in infected skin wounds. The duo showed synergistic effects by inhibiting planktonic MRSA growth and decreasing bacterial adhesion to the wound surface, thereby enhancing treatment efficacy for MRSA-infected wounds (17). Research conducted in animal models and *in vitro* systems encompasses a broad spectrum of investigations, yet evidence derived from human randomized controlled trials (RCTs) remains relatively limited.

### Promoting wound repair and tissue regeneration

Cell death can be classified into type I programmed cell death (apoptosis) and type II programmed cell death (autophagy). Type I programmed cell death, primarily apoptosis, is characterized by cell membrane contraction and nuclear fragmentation. Cao et al. concluded that high glucose inhibits cell proliferation and migration while inducing apoptosis through ROS-dependent activation of JNK and p38 MAPK signaling pathways. In contrast, PRP-Exos prevents high glucose-induced apoptosis by activating the PDGF-BB/JAK2/STAT3/Bcl-2 signaling pathway (18). Autophagy is a highly conserved cellular process responsible for degrading and recycling damaged proteins, organelles, and pathogens. It plays a critical role in the pathogenesis of various diabetic complications (19). Wound healing in the diabetic foot is a continuous dynamic process in which autophagy plays a crucial role, participating in every stage of the healing process. During the initial inflammatory phase, autophagy activation contributes to the polarization of macrophages in the PRP to the M2 phenotype, which plays an important role in immunosuppression and promotion of tissue repair. During the proliferative phase, autophagy is triggered by cellular hypoxia in wounds, which is primarily associated with the regulation of hypoxia-activated protein tyrosine phosphatases and AMPK signaling in endothelial cells (20). In the final remodeling stage, autophagy promotes wound angiogenesis of endothelial cells as well as the differentiation, proliferation and migration of keratinocytes and fibroblasts, facilitating the completion of wound repair and tissue reconstruction. Additionally, experimental findings demonstrated that an increase in LC-3 protein expression, accompanied by a higher LC-3II/LC-3I ratio, indicates an enhanced in autophagic response in PRP-treated diabetic foot ulcer patients (21). The yellow box in **Figure 1** illustrated the mechanism by which PRP promotes wound repair and tissue regeneration.

Ferroptosis is a newly identified form of programmed cell death that is distinct from autophagy and apoptosis. It has been found that GPX4 and SLC7A11 are two key factors associated with the upstream signaling pathway of ferroptosis. Excessive production of ROS in a high-glucose environment reduces the content and activity of GPX4, leading to the accumulation of lipid peroxides and thus leading to lipid peroxidation in the cell membrane and the consequent damage. Ultimately, this results in the occurrence of diabetic foot repair disorders. Al-PRP promotes angiogenesis and repair in ulcer wounds by regulating the expression of ferroptosis-related signature factors. The underlying mechanism involves increasing the protein expression of GPX4 and SLC7A11, promoting the regeneration and repair of skin tissues and vessels in ulcer wounds, thereby accelerating wound healing (22).

### Promotes tissue repair and regeneration

#### Reconstruction of microcirculation in the DFUs

Abnormalities in angiogenesis and extracellular matrix remodeling contribute to the impaired wound healing process in diabetes. Firstly, when PRP is applied to treat diabetic foot wounds, the high concentration of platelets in PRP rapidly aggregates at the wound site, promoting vasoconstriction and forming platelet thrombi at the damaged vessels. Secondly, platelets activate the coagulation system, forming a fibrin network that serves as a scaffold for wound healing. Simultaneously, they release various growth factors, including platelet-derived growth factor (PDGF), transforming growth factor (TGF), insulin-like growth factor, epidermal growth factor (EGF), and vascular endothelial growth factor (VEGF) (23). Neovascularization and restoration of local perfusion rely on the proliferation and migration of vascular endothelial cells, a process strongly driven by VEGF signaling. VEGF upregulates factors such as SDF-1ɑ and its receptor CXCR4, enhancing endothelial chemotaxis and recruitment. Through these pathways, VEGF promotes endothelial infiltration into extracellular matrix-like scaffolds (e.g., collagen gels), supports angiogenesis, and indirectly facilitates osteogenesis by improving vascular supply, delivering progenitor cells, and modulating osteogenic signaling in the healing environment (24). VEGFR exists in several isoforms (primarily VEGFR-1/Flt-1, VEGFR-2/KDR, and VEGFR-3). Among VEGF ligands, VEGF-A is the key driver of angiogenesis in chronic wounds, such as diabetic foot ulcers, mainly via VEGFR-2 signaling to stimulate endothelial proliferation, migration, and permeability (25, 26). In addition, PDGF released from platelets, and secreted by activated macrophages, serves as a crucial mitogenic factor. It plays a role in promoting mural cell remodeling and vascular maturation (27, 28). PRP delivers a spectrum of growth factors that directly and indirectly drive neovascularization, boost cellular proliferation, and collectively hasten wound closure.

In an *in vitro* healing assay, the interaction between platelets and certain plasma proteins in al-PRP strongly stimulates endothelial cell sprouting and induces angiogenesis (29). Animal studies show that wounds treated with lyophilized activated leukocyte−rich PRP (al−PRP) exhibit markedly increased neovascularization compared with controls. This effect is partly attributable to the high VEGF content in PRP, which promotes endothelial proliferation and enhances vascular permeability to regulate vasculogenesis and angiogenesis. In addition, multiple growth factors in PRP act synergistically to further drive vascular formation (30).

#### Promotion of fibroblast proliferation and extracellular matrix synthesis in the DFUs

PRP plays a key role in the formation of the extracellular matrix (ECM), a three-dimensional polymer network composed of various proteins and other components. The ECM not only provides support for the tissue but also facilitates cell adhesion and migration, both of which are critical for the wound healing process. Matrix metalloproteinases (MMPs) are essential for ECM remodeling. Matrix metalloproteinases (MMPs) degrade damaged ECM components, facilitate cell migration, and promote the formation of new tissue. These processes are essential for wound closure and effective tissue repair (31). PRP-enriched EGF, in combination with up-regulated EGFR, promotes DNA and RNA repair, enhances protein synthesis, and stimulates epithelial cell growth, playing a vital role in wound repair. The TGF-β/Smad signaling pathway may induce the onset of epithelial mesenchymal transition by regulating fibroblast proliferation, migration, and by promoting the expression of collagen formation. Simultaneously, the application of PRP reduced the expression level of TNF-α and stimulated the production of substantial collagen fibers, thereby promoting the healing of chronic wounds in diabetic foot. Studies have shown that an increased release of endogenous MMPs inhibitor (TIMP) from the stroma results in inhibition of MMPs synthesis. A decrease in the MMPs/TIMP ratio prolongs the degradation of the trabecular cellular matrix, extracellular matrix, and growth factors, thereby creating a favorable environment for wound healing (32).

In a wound model of diabetic mice treated with al-PRP, Masson trichrome staining showed increased collagen homogeneity and density, while nuclear staining indicated a significant increase in the number of proliferating cells. *In vitro*, the combination of micronized allogeneic dermis and al-PRP stimulated fibroblast. *In vivo*, al-PRP alone or combination with micronized dermis increased wound tissue hemostasis and proliferation (33).

### Regulation of peripheral nerve in the DFUs

The high concentration of growth factors in PRP not only stimulates cell proliferation but also significantly inducing the synthesis of neurotrophic factors. Among these, VEGF plays a critical role in promoting neuronal survival and axon growth. IGF-1 stimulates protein synthesis in neurons, glial cells and Schwann cells. It acts synergistically with VEGF to promote neuronal axon growth. The EGF receptor and its ligands play a role in regulating the response to nerve injury. PDGF promotes the proliferation of human adipose-derived stem cells through activation of the ERK1/2, PI3K/Akt, and JNK signaling pathways, thereby contributing to nerve regeneration. In addition, the high concentration of growth factors in PRP significantly enhances the proliferation and migration of Schwann cells. These Schwann cells connect with nerve stumps to form Büngner’s bands, facilitating axonal regeneration by secreting various active substances, including neurotrophic factors. During the mature stage of nerve regeneration, Schwann cells differentiate into myelin sheaths. Additionally, brain-derived neurotrophic factor (BDNF), present in PRP even at low concentrations, promotes neuronal survival and differentiation while contributing to the maintenance of vascular stability (34). The regulation of peripheral nerves has beendescribed in the gray box of Figure 1.

BDNF and Krox20 are genes associated with nerve regeneration and repair of damaged peripheral nerves. The increased expression of BDNF and Krox20 following al-PRP intervention indicates that al-PRP plays a significant role in enhancing nerve regeneration, particularly in cases of axonal degeneration (35). The regulation of peripheral nerves has been described in the gray box of **Figure 1**.

### Necessity, efficacy and safety of al-PRP in the DFUs

#### Necessity of al-PRP in the DFUs

Diabetic foot ulcers are frequently linked to peripheral neuropathy, vascular dysfunction, inadequate tissue perfusion, microcirculatory impairment, an accumulation of senescent cells, and reduced proliferative capacity of resident cells. This state is marked by attenuated cellular responsiveness to signaling cues. When compounded by external insults—such as trauma, infection, or sustained pressure—it predisposes to refractory wounds and the development of chronic, non-healing ulcers. Delayed healing in diabetic foot wounds markedly elevates the risk of soft tissue and osteal infection, thereby increasing rates of amputation and mortality. These risks underscore an urgent need for therapies that accelerate wound closure and support tissue repair and regeneration. The application of au-PRP is also contraindicated in a significant subset of patients due to comorbidities including hematological disorders, active severe infection, malignancy, and end-organ dysfunction. Practical limitations, such as the procedural costs and logistical challenges of point-of-care preparation, further impede its broad clinical adoption. As a result, the advantages of al-PRP become increasingly evident. A significant safety advantage of al-PRP lies in its local mode of application, which typically prevents entry into the systemic circulation and minimizes the risk of immunogenic reactions. The activation process induces structural and molecular modifications in the platelets, further attenuating their immunogenic potential. Furthermore, al-PRP is fully degraded and absorbed at the application site within weeks, effectively precluding the risk of a chronic immune response. The sourcing of allogeneic blood from regulated blood banks, which adhere to rigorous screening and collection standards, additionally mitigates the risk of transfusion-transmitted infections. Beyond its favorable safety profile, al-PRP exerts potent regenerative effects by promoting key wound healing processes, including angiogenesis, fibroblast proliferation, and collagen deposition. From a clinical standpoint, al-PRP offers considerable logistical and economic advantages. Its off-the-shelf availability streamlines preparation, enhances cost-effectiveness, and facilitates efficient integration into standard wound care protocols (36).

### Efficacy and safety of al-PRP applied to the DFUs

#### Significantly superior to normal dressings

Twenty-four healthy dogs were selected and randomly divided into two groups to establish an abdominal wall defect repair model. The control group received repair using only Damour, while the other group underwent repair with Damour combined with al-PRP. These results showed that dogs treated with al-PRP not only had no hernia recurrence but also exhibited significantly more neovascularization and milder intra-abdominal adhesions. Histological and molecular evaluations further confirmed that after al-PRP treatment, there were significant improvements in collagen deposition and neovascularization, as well as the overexpression of genes related to angiogenesis and myofibroblast proliferation (37). In a controlled clinical trial comprising 100 subjects, wounds treated with al-PRP demonstrated markedly accelerated healing rates, reduced time to closure, and superior wound contraction compared to those receiving conventional topical fibrinogen and thrombin therapy. These findings suggest that al-PRP may promote a more robust and efficient regenerative process (38). In another study, a 20-week follow-up of 24 patients treated with al-PRP and conventional wet dressings revealed a significant reduction in the longitudinal and transverse diameters of the wounds in the al-PRP group compared to the control group. By the end of the follow-up period, the healing rate in the al-PRP group was significantly higher, and the wounds healed at a noticeably faster pace compared to the control group (39). Further studies indicated that al-PRP accelerated the healing of foot wounds even at a 6-month follow-up, with no significant differences based on the subjects’ age, gender, blood pressure, smoking or non-smoking status (40). A meta-analysis revealed that the use of al-PRP resulted in a significantly higher rate of complete wound healing in diabetic foot ulcers compared to the control group (OR: 6.19; 95% CI: 2.32–16.56; *P* < 0.001). Importantly, no increase in adverse effects was observed, highlighting its safety and efficacy (41). The findings from the study suggest that al-PRP offers significant advantages in the clinical management of diabetic foot compared to ordinary dressings.

#### Equivalent results compared to au-PRP

In the animal experiment, 24 healthy adult healthy white rabbits were selected and randomly divided into four groups. One group served as al-PRP donor, while the remaining three groups were designated as experimental groups. In the experiment, the surgical wounds on the left side of the rabbits were treated with saline, while the right side was treated with Au-PRP, Al-PRP, and xenogeneic PRP, respectively. The wounds were monitored and evaluated over a period of 17 days. The results demonstrated that the wounds on the right side achieved complete re-epithelialization, with a significantly higher percentage of wound contraction compared to the contralateral side (42). To evaluate the clinical applicability of autologous leukocyte- and platelet-rich plasma (al-PRP), a study enrolled 75 subjects who received either al-PRP or au-PRP for wound management. He et al. reported no significant difference in platelet concentration between the two treatment groups, with both significantly accelerating ulcer healing. This trial was the first to demonstrate the feasibility of substituting au-PRP with al-PRP in the treatment of DFUs confirming both the efficacy and safety of al-PRP as a comparably effective alternative. Furthermore, within the initial two weeks of treatment, the al-PRP group exhibited a significantly higher rate of granulation tissue proliferation compared to the au-PRP group, indicating a promotive role of al-PRP in the early phase of wound granulation (43).

Although there are numerous animal studies and *in vitro* studies, evidence from human randomized controlled trials (RCTs), as well as large-scale, multicenter trials, is relatively limited. There is an urgent need for standardized preparation procedures for al-PRP, multicenter RCTs, and cost-effectiveness studies.

### Method of preparation and application of al-PRP

The preparation methods of PRP have not yet been standardized. Currently, there are several methods for its preparation: One-step centrifugation method, Two-step centrifugation method, Manual density gradient centrifugation method and the Automated kit-based method. The characteristics of different preparation methods are described in the **Table 1**.

**Table 1:** Comparison of different PRP preparation technologies.

PRP preparation methods	Centrifugal force (×g)	Centrifugation time (min)	Platelet concentration (×10^9^/L) that of whole blood	Disadvantages
One-step centrifugation method	800–120	8–15	1.5–3	1. Low platelet recovery rate 2. Poor concentration 3. High risk of red blood cell contamination
Two-step centrifugation method	1st centrifugation: 1500–2000; 2nd centrifugation: 800–1200	1st: 10–15; 2nd: 5–10	3–6	1. High risk of red blood cell contamination 2. High dependence on operator proficiency 3. Increased contamination risk
Manual density gradient centrifugation method	400–600 (for gradient formation); 800–1000 (for platelet separation)	20–30	4–8	1. Complex operation and high reagent cost 2. Long processing cycle 3. Potential medium residue
Automated kit-based method	900–1500	10–20	2.5–5	1. High cost 2. Limited parameter flexibility 3. Batch-to-batch variability of kits

**Figure 2:**
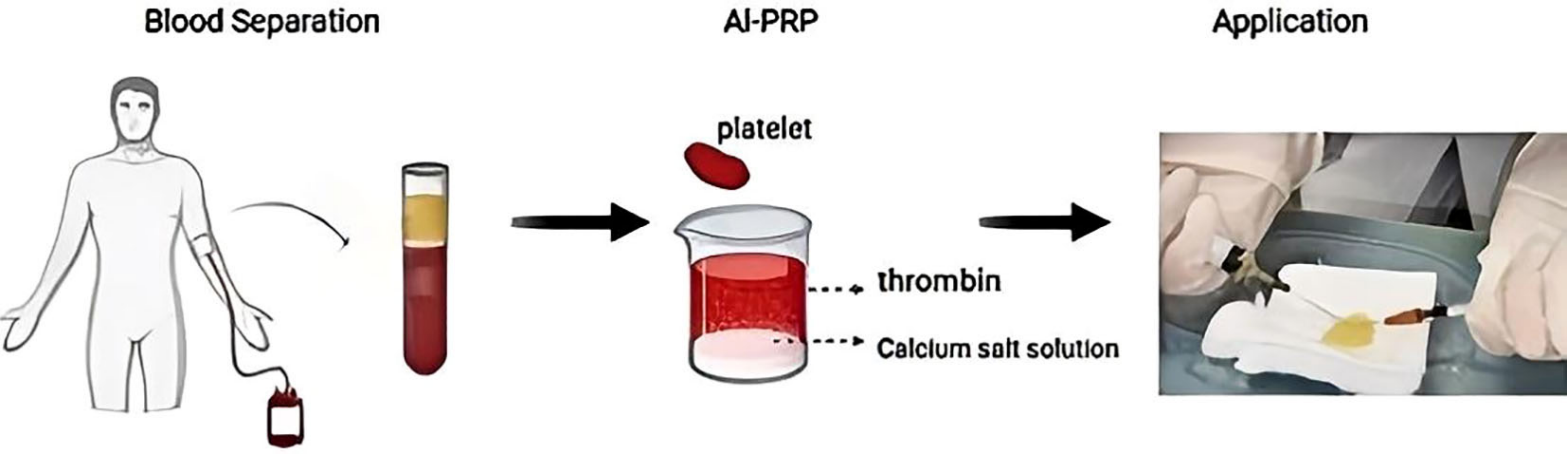
Brief flow chart of platelet-rich plasma preparation.

Our diabetic foot center has established a specific preparation protocol (**Figure 2**), and the preparation of al-PRP differs slightly from that of au-PRP. For al-PRP, Type AB healthy blood donors are selected, and a single platelet treatment volume of approximately 250–300 ml is obtained using blood center equipment. Platelet plasma is divided into 4.5 ml portions and frozen at −20°C for later use. Procoagulant is made by mixing thrombin and a calcium salt solution in a specific ratio, with each 0.5 ml portion also frozen at −20°C. Thawed platelet plasma and the procoagulant are then mixed at a 9:1 ratio to form platelet gel.

Despite the variety of preparation methods, a standardized quality evaluation system for PRP has yet to be established. This calls for cross-disciplinary collaboration among clinical, testing, and materials engineering departments to address this need and improve PRP preparation standards, thereby guiding clinical translation.

### Indications and contraindications for the use of al-PRP in the DFUs

The guidelines recommend platelet concentrate products as a safe and effective therapy for chronic wounds, including vascular ulcers and pressure ulcers. These products promote granulation tissue growth and re-epithelialization in chronic wounds, making them a preferable option compared to traditional conventional wound treatment (44). Al-PRP is mainly applied to wounds where necrotic tissue has been removed, tissue hypoperfusion has been addressed, and infection is under control. Contraindications mainly include: (1) hemoglobin level lower than 10 g/dL; (2) the presence of a tumor or metastatic disease in the wound; (3) hemodynamic instability; (4) sepsis, septicemia; (5) severe local infection. Although extensive literature exists on the application of al-PRP, the absence of large-scale, multi-center, prospective, and high-quality randomized controlled trials (RCTs) highlights the need for further refinement. Specifically, the extraction, preparation, and application protocols for al-PRP require standardization and strengthening to ensure consistency and reliability in clinical practice.

### Practical application of al-PRP in the DFUs

A 61-year-old male patient with diabetes mellitus (disease duration: 19 years) was admitted to the Diabetic Foot Center of our hospital due to a heel ulcer caused by chapping, which had failed to heal for 4 months. At the initial stage of the lesion, there was an ulceration with a diameter of approximately 1 cm, accompanied by exudation. Subsequently, the ulceration enlarged to a 3 cm × 3 cm wound. The patient was classified as Grade 1 according to the Wagner Classification System. Despite receiving routine treatments such as dressing changes and antibiotics at another hospital, the ulceration still failed to heal.

**Figure 3:**
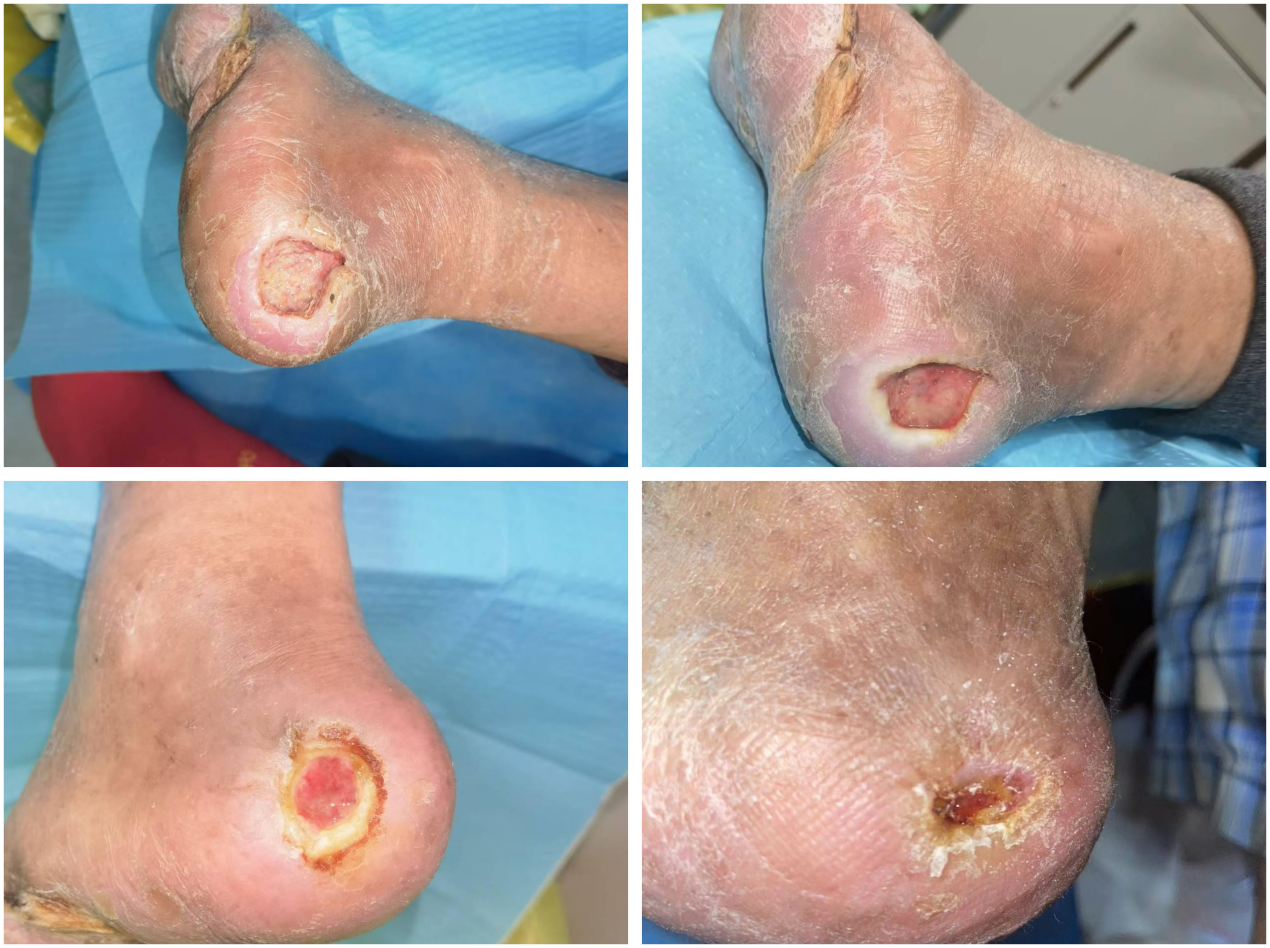
Practical application of platelet-rich plasma in diabetes foot ulcer.

Al-PRP was applied three times a week for four weeks, during which significant wound shrinkage was observed, ultimately leading to healing. Below are the recorded photos documenting the progress. The patient’s consent was obtained for this case. This process is illustrated in **Figure 3**.

## Conclusion

In summary, al-PRP technology plays a role in the treatment of diabetic foot ulcer wounds. However, clinical studies focusing on al-PRP and its combined treatments remain relatively limited, and its full potential has yet to be realized. Therefore, further multidisciplinary research is essential to advance the understanding and application of al-PRP, with the goal of establishing a more comprehensive and effective clinical treatment framework.
